# Interpersonal psychotherapy (IPT) for late-life depression in general practice: uptake and satisfaction by patients, therapists and physicians

**DOI:** 10.1186/1471-2296-8-52

**Published:** 2007-09-13

**Authors:** Digna JF van Schaik, Harm WJ van Marwijk, Aartjan TF Beekman, Marten de Haan, Richard van Dyck

**Affiliations:** 1 GGZ Buitenamstel and Department of Psychiatry, Institute for Research in Extramural Medicine (EMGO), VU University Medical Center, Amsterdam, the Netherlands; 2 Department of General Practice, Institute for Research in Extramural Medicine (EMGO), VU University Medical Center, Amsterdam, the Netherlands

## Abstract

**Background:**

Interpersonal Psychotherapy (IPT) is recommended in most depression treatment guidelines and proved to be a suitable treatment for elderly depressed patients. Despite the favorable results of IPT in research populations, the dissemination to general practice is surprisingly limited. Little is known about uptake and satisfaction when this therapy is introduced into real-life general practice.

**Methods:**

Motivation and evaluation of patients, GPs and therapists were recorded and organizational barriers described alongside a randomized controlled trial. IPT, given by mental health workers, was compared with usual general practitioner (GP) care. Included were patients (≥55 years) who met the DSM-IV criteria for major depressive disorder.

**Results:**

Patients were motivated for the psychotherapy intervention: of the 205 eligible patients, 143 (70%) entered the study, and of the 69 patients who were offered IPT, 77% complied with the treatment. IPT proved to be an attractive therapy for patients as well as for therapists from mental health organizations. General practitioners evaluated the intervention positively afterwards, mainly because of the time-limited and structured approach. Organizational barriers: no IPT therapists were available; an IPT trainer and supervisor had to be trained and training materials had to be developed and translated. Additionally, there was a lack of office space in some general practices; for therapists from private practices it was not feasible to participate because of financial reasons. IPT was superior to usual care in patients with moderate to severe depression.

**Conclusion:**

As we succeeded in delivering IPT in primary care practice, and as IPT was superior to usual care, there are grounds to support the implementation of IPT for depressed elderly patients within general practice, as long as the practices have room for the therapists and financial barriers can be overcome. Consolidation may be achieved by making this intervention available through practice nurses or community psychiatric nurses who deliver IPT as part of a more comprehensive depression management program.

## Background

Depression among elderly primary care patients is common. Of the older patients who visit a primary care clinic 5–10% has a depressive disorder [1,2]. Depression causes suffering and is associated with serious disability, reduced quality of life and general functioning. The course of depression is often chronic or recurrent [3,4]. Delivering effective treatments is of major importance for this group of patients.

Antidepressant drugs and some forms of psychotherapy are both considered to be *evidence based *therapies, as they were effective in older secondary care patients [5]. Some forms of psychotherapy have also proved to be effective in midlife primary care patients [6], but research on older primary care populations is limited [7]. Recently, it has been demonstrated that depression treatment for elderly primary care patients is effective, when a collaborative care model is used in which the primary care physician is supported by a mental health worker in carrying out guideline-driven depression care, including optimal drug therapy, intensively monitoring of drug use, and when indicated, delivering easily accessible evidence based forms of psychotherapy such as IPT or Problem Solving Therapy [8,9]. However, it has consistently been found that the majority of depressed elderly patients in primary care are likely to pass undiagnosed and untreated, with negative mental and physical health consequences [10]. If depression treatment is started, usually antidepressants are offered as first choice treatment. Psychotherapy may be indicated for patients who do not benefit from antidepressant drug treatment or who are sensitive to side-effects, but evidence based psychotherapy is mostly not available in primary care, and referrals to secondary care are frequently not completed by older patients [11]. Apparently, evidence based psychotherapies have not found their way to general practice.

Interpersonal psychotherapy (IPT) seems to be a suitable form of psychotherapy to be delivered to older primary care patients. Its efficacy has been proved [12], and it has been studied both in older patients [13] and in mid life primary care patients [14]. Furthermore, therapists with different therapeutic backgrounds can learn this therapy relatively easily [15]. To explore the implementation potential of IPT, it is important to study uptake and satisfaction when this therapy is introduced into general practice.

In this paper we describe motivation and evaluation of patients, GPs and therapists as well as organizational barriers to introducing IPT for depressed elderly patients into general practice. We recorded these data while conducting a randomized controlled trial, because these data are only interesting when it has been demonstrated that the intervention is effective. This was not yet the case for IPT in elderly depressed primary care patients. In our effectiveness trial, IPT provided by mental health workers was compared with care as usual provided by the general practitioner (GP). The effectiveness results of our study have been detailed in a separate paper [16]. At the end of this paper we integrate the effectiveness results with the findings described in the current paper and discuss whether there are grounds to support the dissemination of IPT for elderly patients in real-life general practice.

## Methods

The study was conducted in 12 general practices in Amsterdam and surroundings, based on a protocol approved by the Medical Ethics Review Committee of the VU University Medical Center in Amsterdam.

### Patients

The patients were recruited by means of a two-stage screening procedure. Our research assistant visited the general practices about once a month to collect names and addresses from the computerized databases of the GPs, of patients of 55 years or older, who had visited the practice in that last month. These patients were sent a letter on behalf of their general practitioner, in which they were asked to fill in a screening questionnaire, the 15-item Geriatric Depression Scale, GDS-15 [17], and return it to our research center. Patients, who scored 5 or more on the GDS-15, were contacted by telephone by one of the research assistants and were invited to participate in the diagnostic procedure. The diagnostic instrument used to assess depressive disorder was the mood module of the PRIMary care Evaluation of Mental Disorders, PRIME-MD [18]. Exclusion criteria were: receiving treatment for depression at the time of screening, insufficient command of the Dutch language or severe cognitive impairment (MMSE < 18). Those who signed informed consent were randomly allocated to IPT or CAU. An independent research assistant performed randomization per practice at the patient level by using a table of random numbers.

### Therapists and general practitioners

General practitioners cannot deliver IPT themselves because this therapy is very time-consuming and not part of GPs vocational background. In the Netherlands as in many other countries, general practitioners often collaborate with psychologists and counselors from private practices or with psychiatric nurses and psychologists from the regional organizations for mental health care. For practical reasons we chose to approach the organizations for mental health care as a starting point, because they often have networks of general practitioners with whom they collaborate (which facilitated the recruitment of GPs), and we assumed that they were able to make enough therapists available for the project (which made training and supervision of therapists, who are working in one organization, more efficient). For this same last reason, we also approached group private practices for psychotherapy.

One of the project members (DvS) was trained as an IPT trainer and supervisor. We used the Comprehensive Guide to IPT and additional literature on IPT for the elderly as a basis for developing training materials in Dutch [15,19,20]. Training in standardized IPT was given in a 2-day course, followed by group supervision sessions (four therapists per group) every two weeks during participation in the trial.

### Intervention

IPT is a structured, time-limited therapy for depression. In the initial phase of the treatment the depressive symptoms are explored and psycho-education about depression is given. The interpersonal context of the patient is explored and the depressive symptoms are linked to recent interpersonal events. There are four possible treatment focuses to be distinguished: complicated grief, interpersonal conflict, role-transition and interpersonal deficit. One of these focuses is chosen. The nature of this specific interpersonal event is explored and accompanying emotions are clarified. The patient is supported in considering and working out possible solutions. It is assumed that once the patient has gained mastery over one problem area this effect will generalize to other areas. During the last sessions, the therapy is evaluated, and attention is paid to the prevention of relapses.

For use in general practice the number of IPT sessions was reduced from the usual 12 to 10. The structure of the IPT we delivered was identical to the original protocol; only the number of sessions in the treatment phase was reduced. We assumed that with this smaller number of sessions dropout would be less likely. We also assumed that a less intense type of treatment would be sufficient, because of the usually less severe nature of major depressive disorders in general practice [21]. The aim was to complete the therapy within five months.

### Uptake and satisfaction

Data concerning the recruitment of patients, therapists and GPs were collected. The therapists recorded the number of IPT sessions that were completed, dropout rates and dropout reasons. Additionally, all participants evaluated the intervention. At six months follow-up, patients were asked to evaluate the intervention by means of the 8-item Client Satisfaction Questionnaire (CSQ, of this measure the mean item score is usually presented, ranging from 1 to 4 [22]). At the end of the project, the therapists and the participating GPs were sent a questionnaire containing specific and open-ended questions about the intervention. Furthermore, organizational barriers and facilitating factors were recorded during the project by the researcher, research assistants and therapists.

## Results

### Motivation of the participants

#### Patients

The GDS-15 was presented to 6.719 patients. Of them, 4.143 patients (62%) could be fully screened (GDS-15 and PRIME-MD if the GDS-15 score was ≥5). Of the 205 eligible patients with current major depression, 143 (70%) gave informed consent and therefore had a positive or at least neutral attitude towards IPT. The research population consisted of the 69 patients who were allocated to the IPT condition. Table 1 presents the characteristics of these patients. To assess depression severity we used the Montgomery Åsberg Depression Rating Scale (MADRS, range 0–60, higher scores indicate higher severity of depression. The mean score of 19.4 means that on average patients had mild depressive symptoms. [23]) The mean number of treatment sessions was eight. Of the 69 patients who were offered the therapy, one failed to initiate and 47 (68%) completed 10 sessions. Six patients (9%) terminated the therapy earlier while the therapist agreed that they did not need to continue any longer. Thus, at least 77% of the patients were compliant with the therapy. Other reasons for dropout were: somatic diseases (4), organic brain syndrome (1), death (1), long-time stay in other part of the country (2), no motivation (5), and reason not clear (3).

**Table 1:** Basic characteristics of patients allocated to the IPT intervention

Characteristics	IPT (n = 69)
**Sociodemographic**	
Mean (SD) age, years	68.4 (8.1)
Female (%)	48 (70)
Married or living together (%)	33 (48)
Level of Education (%)	
Low	23 (33)
Intermediate	27 (39)
High	19 (28)
**Clinical**	
MADRS, mean (SD)	19.4 (7.9)
CGI-s, median (SD)	3.3 (1.3)

Note: MADRS: Montgomery Åsberg Depression Rating Scale; CGI-s: Clinical Global Impression severity scale.

#### General practices

Managers of four specialist mental health centers contacted general practices that were part of their network. Practices that could be reached within half an hour from the office of the mental health workers or psychotherapists were contacted by telephone and invited to participate. Of the 18 general (mostly group) practices that were approached, six refused. Lack of available office space for the therapists was mentioned as the main barrier. One GP objected to the screening procedure and one saw no need for this intervention.

#### Private psychotherapy practices, specialist mental health centers and mental health workers

The two psychologists' private practices that were approached, were interested in IPT, but could not join the project, because it was not feasible for them to travel to the general practice to deliver the psychotherapy. Specialist mental health centers were motivated to provide therapists for the project, because transfer of expertise from specialized care settings to the primary care setting has high priority in Dutch health policy. In addition, in the Dutch system, these centers have a duty for outreaching care and have the possibility for remuneration of travel expenses, while private practices have not. Four centers provided therapists. The geriatric teams of these organizations received training in IPT, and it turned out that the majority of therapists were interested in joining the research project. Nine psychiatric nurses and six psychologists participated in the study. Of these therapists 10 were female. Their mean age was 47 yrs (SD 7.8). All therapists had worked for more than five years in mental health care, and 13 had two or more years of experience in working with elderly patients. Of the psychologists, one had a psychodynamic background; four were trained in cognitive behavioral therapy, and one in family therapy. The psychiatric nurses had not been trained before in a specific psychotherapeutic approach. Supervision was provided at the workplace of the therapists and was well attended.

### Evaluation of the intervention

#### Patients

Patient satisfaction was measured by means of the CSQ-8, which was completed by 54/69 patients. The mean item score on this scale was 3.0 (SD 0.6), which represents a positive evaluation.

#### General practitioners

All of the 22 GPs, who had one or more of their patients treated with IPT, returned the evaluation questionnaire. The questionnaire started with two open ended questions ("What were positive/negative aspects of delivering IPT transmurally to depressed elderly patients within you practice?") The following positive aspects were mentioned more than once: 11 GPs gave positive comments regarding the IPT intervention as such (time-limited, practical, patient-friendly and structured); nine mentioned the easy access of the intervention; four GPs mentioned that they felt supported in the depression care for the elderly by this intervention. Negative comments that were mentioned more than once: three GPs mentioned that they had had hardly any contact with the therapist; two GPs thought that screening was not the right way to select patients, but that referral should be done by the GPs; two GPs found the intervention time-consuming. On a specific question about the usefulness of delivering IPT within the practice, all but one of the GPs thought that this intervention lowered the barrier to providing adequate care for a group of elderly patients, who do not want to be referred to specialist mental health facilities. In the last question GPs were asked whether they would use this intervention if it would be available after the end of the research project (yes/no). Again, all but one was positive.

#### Therapists

The participating therapists were positive about working within the general practice. Most of them were used to doing outreaching work, and the visits to the GP could be integrated well into their schedule. Of the 15 therapists, 11 reported that they occasionally needed extra time and flexibility to find office space, and one found this a very unpleasant complication. Therapists were positive about the structure and strategies provided by the IPT protocol. The focus 'role transition' was chosen in 60% of the cases. The main subject of this focus was the transition from being a healthy person to a person who is restricted by physical disabilities. Role dispute with an important other was the treatment focus in 22% of the cases. Grief and interpersonal deficit were chosen less often. In patients with mild depression it was difficult to discuss the depressive symptoms as part of a depressive illness, as is described in the original IPT protocol. In an early stage of the project, it became clear that this approach was not suitable for these patients, because they did not recognize themselves in the description of having a depressive *illness*. The therapists were also convinced that speaking about an illness was inappropriate for these patients. Therefore, the therapists linked the interpersonal problems to the complaints of the patients instead of to a depressive disorder.

### Effectiveness of the IPT intervention in our trial

The effectiveness data are described in a separate paper [16], but we will summarize them here, because findings regarding uptake and satisfaction are only relevant when an intervention has proved to be effective. All patients who entered the study had a DSM-IV diagnosis of major depressive disorder according to the PRIME-MD. There was no difference between the intervention and control group regarding baseline sociodemographic and clinical characteristics. In the IPT group, 51% of the patients had no diagnosis of depression at six months follow-up, compared with 34% in the control group (X^2^_[df]= _= 4.21_[1]_; p = 0.04). In a post hoc analysis, we stratified the sample in patients who had a mild depression at baseline and in patients who had a moderate to severe depression (MADRS cut off score 21). In the group with moderate to severe depression 54% of the patients in the IPT group had no diagnosis of depression anymore at six months, compared with 26% in the control group (X^2^_[df]= _= 4.75_[1]_; p = 0.03). In the group of patients with mild depression these percentages were 49% and 40% respectively (X^2^_[df]= _= 0.63_[1]_; p = 0.43).

## Discussion

In this paper we explored uptake and satisfaction when IPT was introduced for depressed elderly patients in general practice. Some issues are generic and have to be overcome before any psychotherapy intervention can be delivered within general practice, others are specific to IPT. With regard to organizational barriers to providing evidence based psychotherapy within general practice: Mental health organizations joined the project, because the transfer of expertise from specialized care to the primary care setting has high priority in Dutch health policy. In the Netherlands, the distances from the secondary care offices to the general practices can be relatively easily overcome, and in the majority of the general practices office space can be arranged for the mental health workers. General practitioners felt supported by the mental health workers in the treatment of the depressed elderly. With regard to IPT: Although there were no trained IPT therapists available, it was feasible to organize IPT training and supervision. Mental health organizations were willing to give their therapists time to be trained in IPT, because they wanted to support the dissemination of this short term, evidence based form of psychotherapy within their organization. IPT proved to be an attractive therapy for the older patients in real-life practice and for the therapists. General practitioners were positive about the structured and clear approach of IPT.

IPT was superior to usual care in patients with moderate to severe depression, not in patients with mild depression. Our screening procedure, using the PRIME-MD as the primary measure to diagnose major depression, yielded many patients with only mild symptoms (57%). To select patients for the intervention, we concluded that a measure to assess depression severity should be added. When delivered only to patients with moderate to severe depression, the number of sessions should most probably be increased as Shapiro et al. have found that patients presenting with severe depression improved substantially more after 16 than after 8 sessions of IPT [24]. The overall effectiveness of the depression treatment can of course be improved when patients can choose or switch between interventions, and combination therapies can be given, as is done successfully in several collaborative care depression management programs [8,9].

### Comparison with other studies introducing IPT in primary care

In three other studies IPT was delivered in primary care settings. [8,14,25]. The recruitment procedures and the target populations differed (regarding age range), which makes it difficult to compare for instance the motivation for IPT. With regard to dropout rates: In the study of Schulberg 50% dropped out in the acute phase, in the PROSPECT study this percentage was 38% compared with 32% in our study. Browne defined adherence as attending 80% of the sessions offered. According to this definition 20% dropped out, compared to 26% in our study, when we use this same definition. Thus, the dropout rates in our study were relatively low, but the proposed number of sessions was also lower than those in the other studies (10 instead of 12 to 16). In the three studies mentioned no information is given about how the therapists were recruited and how they evaluated IPT. No data about possible organizational barriers and facilitating factors were given.

### Limitations

1. Our findings apply to the situation in the effectiveness trial. Although our trial was carried out in practices that were not affiliated with our university center, and findings were therefore more generalizable than those from efficacy trials, long-term feasibility outside the context of a trial, still needs to be studied.

2. It can be argued that we may not have included a representative group of general practitioners. Indeed, most of the general practices already collaborated intensively with the mental health organizations, and therefore probably had higher than average affinity with mental health problems. However, our findings give an indication that at least a substantial percentage of GPs is interested in this intervention and that, once on board, they evaluate it positively.

3. The way we measured patients' attitudes towards the intervention can be criticized. We conclude that patients were motivated because of the relatively high percentage of eligible patients that wanted to participate and complied with the therapy, and because of the scores on the client satisfaction questionnaire (CSQ-8). Yet, this questionnaire gives only a global impression of how the intervention was received, no specific information regarding the IPT protocol. Moreover, the mean score of the intervention group gives limited information, because we could not compare this mean score with a valuable reference score (as this is unknown for this target population). Furthermore, we were not able to compare the scores of the intervention group with that of the usual care group, because the majority of the patients in the control condition did not receive any depression treatment at all and consequently, did not complete the CSQ.

4. The percentage of eligible patients who were motivated for the intervention (70%) cannot be generalized as such to all older primary care patients, because we were not able to screen the whole target population. Probably, the percentage of refusals would be higher among the depressed non-completers of the screening procedure. Additionally, our population was primarily from (sub)urban regions. In a survey of McKeon, living in a city was positively associated with a preference for psychotherapy [26]. Thus, the estimate of 70% is probably too high, but notwithstanding that we can conclude that from the perspective of many older patients in general practice, IPT is a welcome treatment option.

5. Some findings may not be generalizable to other countries. Patients did not have to pay for their therapy. Therefore, we do not know to what extent financial barriers may influence uptake in real life practice in countries with a different financial organization of the health service. Training and supervision in IPT may also be more difficult to organize in other countries, but there is an excellent treatment manual available that recently has been updated [15]. Moreover, there is an International Society for IPT, which shares knowledge and experiences in IPT research, training and supervision [28].

## Conclusion

We conclude that there are grounds to support the implementation of IPT for depressed elderly patients within general practice, as long as the practices have room for the therapists and financial barriers can be overcome. In the Netherlands, there is an ongoing development of general practitioners being supported by psychiatric nurses in mental health tasks. IPT fits well in the tool-kit of these nurses. This implies that an important gap in the depression care for older patients can be filled. For patients who prefer psychotherapy [27] or who do not benefit from antidepressant drug treatment (alone), IPT is an attractive evidence based treatment alternative, especially when it is delivered selectively. IPT was more effective in moderate to severe depression, and should be reserved for this group of patients. Consolidation may be achieved by making this intervention available through practice nurses or community psychiatric nurses who deliver IPT as part of a more comprehensive depression management program.

Future research should focus on the fine-tuning of the intervention (e.g. optimal number of sessions, comparing IPT with Problem Solving Treatment) and on the feasibility of long-term implementation.

## Competing interests

The author(s) declare that they have no competing interests.

## Authors' contributions

DvS, HvM and AB have made substantial contributions to the conception and design of the study and to interpretation of the data. MdH and RvD have been involved in interpretation of the data and in drafting the manuscript and revising it critically.

All authors read and approved the final manuscript.

## Pre-publication history

The pre-publication history for this paper can be accessed here:


